# Multidisciplinary lifestyle intervention in children and adolescents - results of the project GRIT (Growth, Resilience, Insights, Thrive) pilot study

**DOI:** 10.1186/s12887-020-02069-x

**Published:** 2020-04-20

**Authors:** Hannah L. Mayr, Felicity Cohen, Elizabeth Isenring, Stijn Soenen, Project GRIT Team, Skye Marshall

**Affiliations:** 1 Bond University Nutrition and Dietetics Research Group, Faculty of Health Sciences and Medicine, Bond University, Gold Coast, Queensland Australia; 2 Weight Loss Solutions Australia, Gold Coast, Queensland Australia; 3 Department of Nutrition and Dietetics, Princess Alexandra Hospital, Brisbane, Queensland Australia; 4 Adelaide Medical School, Centre of Research Excellence in Translating Nutritional Science to Good Health, The University of Adelaide, Adelaide, South Australia Australia; 5 Faculty of Health Sciences and Medicine, Bond University, Gold Coast, Queensland Australia; 6 Physiologic Physiotherapy and Sports Medicine Clinic, Gold Coast, Queensland Australia; 7 Nutrition Research Australia, Sydney, New South Wales Australia

**Keywords:** Exercise, Physical activity, Diet quality, Self-concept, Children, Adolescents, Lifestyle intervention, Multidisciplinary

## Abstract

**Background:**

During childhood and adolescence leading behavioural risk factors for the development of cardiometabolic diseases include poor diet quality and sedentary lifestyle. The aim of this study was to determine the feasibility and effect of a real-world group-based multidisciplinary intervention on cardiorespiratory fitness, diet quality and self-concept in sedentary children and adolescents aged 9 to 15 years.

**Methods:**

Project GRIT (Growth, Resilience, Insights, Thrive) was a pilot single-arm intervention study. The 12-week intervention involved up to three outdoor High Intensity Interval Training (HIIT) running sessions per week, five healthy eating education or cooking demonstration sessions, and one mindful eating and Emotional Freedom Technique psychology session. Outcome measures at baseline and 12-week follow-up included maximal graded cardiorespiratory testing, the Australian Child and Adolescent Eating Survey, and Piers-Harris 2 children’s self-concept scale. Paired samples t-test or Wilcoxon signed-rank test were used to compare baseline and follow-up outcome measures in study completers only.

**Results:**

Of the 38 recruited participants (median age 11.4 years, 53% male), 24 (63%) completed the 12-week intervention. Dropouts had significantly higher diet quality at baseline than completers. Completers attended a median 58 (IQR 55–75) % of the 33 exercise sessions, 60 (IQR 40–95) % of the dietary sessions, and 42% attended the psychology session. No serious adverse events were reported. Absolute VO_2_peak at 12 weeks changed by 96.2 ± 239.4 mL/min (*p* = 0.06). As a percentage contribution to energy intake, participants increased their intake of healthy core foods by 6.0 ± 11.1% (*p* = 0.02) and reduced median intake of confectionary (− 2.0 [IQR 0.0–3.0] %, *p* = 0.003) and baked products (− 1.0 [IQR 0.0–5.0] %, *p* = 0.02). Participants significantly improved self-concept with an increase in average *T*-Score for the total scale by 2.8 ± 5.3 (*p* = 0.02) and the ‘physical appearance and attributes’ domain scale by median 4.0 [IQR 0.5–4.0] (*p* = 0.02).

**Conclusions:**

The 12-week group-based multidisciplinary lifestyle intervention for children and adolescents improved diet quality and self-concept in study completers. Future practice and research should focus on providing sustainable multidisciplinary lifestyle interventions for children and adolescents aiming to improve long-term health and wellbeing.

**Trial registration:**

ANZCTR, ACTRN12618001249246. Registered 24 July 2019 - Retrospectively registered

## Background

The increasing prevalence of cardiometabolic risk factors, such as obesity, dyslipidaemia, elevated blood pressure, hyperglycaemia and poor cardiorespiratory fitness during childhood and adolescence adversely affects development, growth, maturation, mental health and quality of life [1–4]. Furthermore, the development of risk factors in childhood significantly increases the likelihood of developing cardiometabolic disease in adulthood and has adverse consequences on premature mortality and physical morbidity [5–7]. The prevention of developing cardiometabolic disease risk factors in childhood is a recognised global priority [4, 8].

During childhood and adolescence, leading behavioural risk factors for the development of cardiometabolic disease include poor diet quality and sedentary lifestyles [9–11]. Recent national survey data in Australian children and adolescents (aged 2 to 18 years) found that intake of discretionary foods contributed to 40% of overall dietary energy intake; where close to three-quarters of the sample exceeded recommend intakes for free sugars and less than 1% met recommended intakes of vegetables [12]. In addition, national recommendations for engagement in physical activity were met by only 30% of these children and adolescents [13].

Lifestyle interventions appropriate for children and adolescents are an important mechanism for improving dietary and/or physical activity habits. Studies of multi-disciplinary interventions in children and adolescents involving both dietary education and physical activity sessions have demonstrated improvements in cardiometabolic outcomes (low-density lipoprotein, triglycerides, fasting insulin, and blood pressure) [14]. Evidence suggests that combined diet and exercise interventions in children and adolescents have greater effects on measures of metabolic health and obesity prevention than single interventions [15, 16].

Previous trials of lifestyle interventions have largely focused on weight management for overweight or obese children and adolescents or prevention of weight gain [17, 18]. However, recent evidence argues the importance of improving cardiovascular health rather than weight in children and adolescents and focussing on promoting a healthy body rather than a slim body [18]. The psychosocial impacts of interventions are also important to consider as self-esteem in childhood may remain stable into adulthood [19]. A recent review of interventions which measured self-esteem changes in children following participation in weight management programs recommended limiting emphasis on weight status change, including parental involvement, and conducting the intervention in a group setting to provide a positive social experience [20]. Self-esteem in children and adolescents may be measured by self-concept scales, which incorporate multiple constructs (e.g. academic, physical, social and behavioural) and are a useful method for elucidating the effect of lifestyle interventions on both global self-esteem as well as its unique dimensions [20].

Healthy eating interventions in schools have demonstrated that experiential learning approaches, such as community gardens, cooking demonstrations, or food preparation activities, were associated with the largest impact on improved diet quality and nutritional knowledge [21]. In addition, a recent review determined that evaluation of lifestyle programs for children and adolescents in non-institutional (e.g. outside of hospital or schools) settings are needed [14, 17].

To meet these needs, Project GRIT (Growth, Resilience, Insights, Thrive), a multidisciplinary lifestyle intervention for sedentary children and adolescents, was developed. Project GRIT involved group exercise training, dietary education, and a psychology session in a non-institutional setting on the Gold Coast, Australia. The aim of this study was to determine the feasibility and effect of Project GRIT on cardiorespiratory fitness, nutrient intake, diet quality and self-concept in sedentary children and adolescents aged 9 to 15 years.

## Methods

### Study design

Project GRIT (Growth, Resilience, Insights, Thrive) was a pilot single-arm intervention study (Australia and New Zealand Clinical Trials Registry: ACTRN12618001249246) reported according to the template for intervention description and replication (TIDieR) checklist [22]. Project GRIT was a 12-week multidisciplinary intervention which aimed “to build skills, knowledge and behaviour to help kids lead healthy and happy lives”, with no cost associated with participation. The intervention involved weekly group-based High Intensity Interval Training (HIIT) sessions, five healthy eating or cooking demonstration education sessions, and one mindfulness and Emotional Freedom Technique (EFT) psychology session. The study site was a private medical centre in the metropolitan location of Gold Coast, Queensland, Australia; where the intervention was delivered both onsite (diet and psychology sessions) and offsite (exercise sessions and one cooking demonstration) at a publicly accessible outdoor recreational park and commercial kitchen, respectively. The study was conducted in accordance with the Declaration of Helsinki [23]. All procedures involving participants were approved by the Human Research Ethics Committee of Bond University (SM02967), with written informed consent obtained from all enrolled participants, a parent/guardian, and the participant’s usual General Practitioner prior to participation. If the participant reported a medical illness during the study which could impact their appropriateness for continued involvement in the exercise training, the participant was required to re-consult their General Practitioner for re-consent regarding exercise participation. If re-consent by the General Practitioner was not obtained, participants could continue in the non-exercise related activities only.

### Participants

Participants for this study were recruited by the Project GRIT coordinator between May and July 2018. The eligibility criteria are listed in Table 1. As this study represented a preliminary analysis in a pilot cohort, a sample size calculation was not performed [24]. Instead, the target sample size was 50 children, which was chosen to reflect resources of the study site and recruitment feasibility. Recruitment methods included: online and social media advertising, newspaper advertising, newsletters distributed to site stakeholders, communication with approximately 70 local General Practitioner medical centres, and broadcasting through a local television news program. The recruitment advertising targeted both children and parents/guardians living across the city of Gold Coast council area. All advertising directed potential participants to the Project GRIT website which asked for their contact details to register their interest, which was followed up by the Project GRIT coordinator to discuss the program and conduct initial eligibility screening. Age, sex and sibling involvement of potential participants were collected from the parent/guardian at screening. The next phase of recruitment of potentially eligible participants was to attend a group information session at the study site, where informed consent was obtained in addition to agreement to a Project GRIT Code of Behaviour and Conduct, and an indemnity form. All participants and their parent/guardians were given an opportunity to consider participation and ask further questions.

**Table 1:** Project GRIT Participant Eligibility Criteria

Inclusion Criteria	Exclusion Criteria
• Aged 9–15 years. • Inactive (self-reported as inactive; no specific criteria applied). • Participant and parent or guardian able to support lifestyle changes and commit to a 12-week program between July – October 2018 with an intention of ≥80% attendance of all Project GRIT sessions.	• Known diagnosis of learning disorder and/or medical condition with which the multidisciplinary Project GRIT staff cannot provide sufficient support for, including: Attention Deficit Hyperactivity Disorder, Autism, Asperger Syndrome, Tourette Syndrome, or Bipolar Disorder. • Known diagnosis of a medical condition which contraindicates high-intensity exercise, including: o Hypertension as defined by systolic and/or diastolic blood pressure ≥ 95th percentile measured upon three or more occasions o History or evidence of cardiac abnormalities or family history of hypertrophic obstructive cardiomyopathy o Hypercholesterolaemia o Chronic disease including but not limited to kidney disease, chronic asthma, diabetes (type I or II) o Orthopaedic or neurological disorder which limits physical activity o Pulmonary disease • Current smoker • Use of steroid medications • Food allergy which would prevent the child from involvement in healthy eating or cooking demonstration sessions

### GRIT intervention

Following screening and attainment of necessary study approvals, each recruited participant was provided with a GRIT t-shirt, visor, and drink bottle. Participants were also provided with a Polar A300 heart rate and activity monitor watch and Polar H7 heart rate sensor chest strap (Polar Electro Oy, Kempele, Finland), which were required to be returned at the close of the project. Participating children and their parents/guardians, Project GRIT staff, and research personnel were not blinded to the purpose of the intervention or data collection measures as the program was intended to be delivered in a usual clinic setting. Attendance was recorded at all intervention sessions. A summary of the scheduling of intervention components across the weeks of the program is provided in Table 2.

**Table 2:** Schedule of the GRIT Intervention Components

Week of program	HIIT Exercise	Diet	Psychology
	*Number of sessions offered*		
1	3	1	
2	3		
3	3	1	
4	3		
5	3	1	
6	3	1	
7	2		
8	3		1
9	2		
10	2	1	
11	3		
12	3		

*HIIT* high intensity interval training

#### Exercise sessions

Research trials have demonstrated that HITT improves cardiorespiratory fitness and cardiometabolic risk markers in children with similar effects to Moderate Intensity Continuous Training (MICT), however, it is more time-efficient [25, 26]. Furthermore, in adults, running using HITT was perceived to be more enjoyable than MICT [27]. A HIIT model was therefore chosen for the exercise sessions in GRIT. The program involved three group sessions of HIIT per week, which each lasted for approximately 30-min and were offered on Mondays, Thursdays and Saturdays. The HIIT sessions were conducted at a local outdoor recreation park (approximately 3 km travel from the medical centre offices). The sessions were implemented by a qualified Athletics Coach and Physical Education Teacher with assistance from an additional supervisor. Parent/guardians attended and supervised each HIIT session which their child attended.

The HIIT component involved intermittent fast running (which aimed for ≥85% of estimated maximum heart rate [HRmax]) for short periods followed by long active recovery periods where the participants were walking or lightly jogging. No exercise equipment was used. Each exercise session also began with a slow run warm up followed by a gentle supervised stretch and finished with an easy 200 m walk. The following interval sets, based on a percentage HRmax, were used in the HIIT sessions, with a gradual progression through the 12-weeks:

- 15-s high intensity activity at ≥85% HRmax with 2.45-min recovery at 50–70% HRmax.
- 30-s high intensity activity at ≥85% HRmax with 4.30-min recovery at 50–70% HRmax.
- 1-min high intensity activity at ≥85% HRmax with 5-min recovery at 50–70% HRmax.

This active recovery zone has been utilised in other HIIT based training protocols in children [28]. For the purpose of determining each participant’s HR recovery zone, HRmax was calculated via a validated age-based equation [29]. From week 2 onwards, all participants were provided weekly make-up session protocols which mirrored what was being done in the group sessions, via email. These were intended for the child to complete in their own time under the supervision of a parent/guardian if they were unable to make a group training session.

#### Heart rate monitoring

During all exercise sessions, either as part of the GRIT program or make-up sessions in a private environment, participants were asked to wear the Polar A300 watch and paired H7 chest strap. During week 1 GRIT exercise sessions, the children were guided on how to correctly wear the chest strap, pair it with their watch and initiate and cease data collection. Participants were also provided their HR recovery zone and guided on using their HR which was displayed in real-time on the Polar watch during the session intervals. Participants were advised not to wear their chest strap during exercise they engaged in outside of the GRIT group and makeup exercise sessions.

#### Healthy eating and cooking demonstration workshops

Three workshops were held which focused on healthy eating and two workshops were held involving a cooking demonstration. All sessions used a weight-neutral and non-diet approach [30] and were interactive with involvement of participants and their parent/guardians. Each healthy eating session and the second cooking demonstration was implemented in small groups (maximum 20 participants) by an Accredited Practising Dietitian (APD) and was held for 30 min. The first cooking demonstration involved two guest chefs and was held for approximately one hour and included all participants. Details of each of the healthy eating and cooking demonstration sessions are provided in Supplementary Materials, Table S1. Briefly, the healthy eating session topics were (1) healthy lunchbox challenge, (2) healthy snack recipe modification, and (3) food for mood. The guest chef cooking demonstration included a healthy breakfast meal and snack. The APD cooking demonstration involved preparation of sushi rolls. Mid-way through the program, parents were also provided with a hard and/or electronic copy of the Australian Dietary Guidelines Healthy Eating for Children brochure, which provides evidence-based recommendations on the amount and types of foods children should be eating for health and wellbeing [31]. Each of the three healthy eating sessions were filmed (capturing the instructing APD only) and a private link to the video of these sessions was sent to all parent/guardians so that any children unable to attend were able to review the material in their own time. Appropriate food safety and handling procedures were followed in the healthy eating and cooking demonstration sessions.

#### Emotional freedom technique and mindfulness workshop

In week 8, the psychologist ran a single 40-min group workshop at the medical centre offices which covered EFT and mindful eating. The EFT component involved instructions on using tapping, which is an alternative behaviour technique to self soothe [32]. These instructions included advised tapping points on the body, a series of tapping steps to follow, and example statements to say out loud whilst undertaking the tapping steps. The psychologist and participants shared situations when tapping could be used as a soothing technique. The children were each provided with a brochure including a summary of these instructions [33] and were encouraged to use tapping as a soothing technique at home or school. EFT was chosen as an adjunct psychological component to the GRIT program as it is simple to teach, able to be delivered in a group setting and has been demonstrated to improve eating habits and self-esteem in adolescents [34]. The mindfulness component focused on eating behaviour techniques, including guided eating meditations and discussions. The eating behaviour techniques focused on attending physical hunger, satiety, taste, and awareness of cues to eat; it also focused on the practice of savouring tastes and textures [35]. During the workshop, the psychologist guided participants through a mindful eating exercise with a raisin.

### Study measures

Study measures have been summarised in Table 3.

**Table 3:** Summary of Study Measures

Study Measure	Timepoints	Explanation	Related intervention component/s
Attendance	All sessions from baseline to 12 weeks	Measure of feasibility	All
Retention	1st exercise session to last attended study session	Measure of feasibility Total days involved and number of participants completing the study versus withdrawal	Program involvement
Adverse events	Reported at any exercise, dietary or psychology session	Measure of feasibility Minor or major Assessed whether unrelated, potentially related or related to the study	All
Satisfaction	12 weeks or at withdrawal	Measure of feasibility Collected via written surveys with Likert scaled questions	All
Heart rate during exercise sessions	Continuously during HIIT group exercise sessions and individual make-up sessions	Guide for participants during exercise sessions to achieve high intensity and recovery heart rate targets Changes in HR across the intervention are a fitness indicator	Exercise sessions
Anthropometry	Baseline	Participant characteristics for weight, height and waist circumference, with calculation of BMI, BMI-for-age percentiles and Z-score	Not a target of any interventions
Maximal graded cardiorespiratory testing	Baseline and 12 weeks	VO_2_peak: Peak oxygen consumption during testing as a measure of maximal exercise capacity. Testing time to reach VO_2_peak measures time to exertion. HRmax: Maximum heart rate measured during exercise testing. A reduction in HRmax over time can indicate improvements in cardiac output. MFO: maximum fat oxidation measure during testing, positively associated with respiratory capacity and training status [36].	Exercise sessions
Australian Child and Adolescent Eating Survey FFQ (Nutrient intake and diet quality)	Baseline and 12 weeks	Total and food-group based Australian Child and Adolescent Recommended Food Scores, measures of diet quality reflecting adherence to the Australian Dietary Guidelines. Estimated daily intake of food groups as a percentage contribution to total energy intake. Estimated macro- and micronutrient intake	Dietary education sessions and cooking demonstrations
Piers Harris-2 Self-concept scale	Baseline and 12 weeks	Global measure of self-esteem. Measures total and domains of behavioural adjustment, intellectual and school status, physical appearance and attributes, popularity, happiness and satisfaction, and freedom from anxiety	All

*HIIT* High Intensity Interval Training, *FFQ* food frequency questionnaire

#### Process evaluation

Attendance of participants was recorded by the project coordinator at each of the program sessions. The withdrawal of participants was recorded, including the date and week of the program and reason, if disclosed. Time involved in the program for participants who withdrew or who were lost to follow up was calculated in days from the date of the first exercise session to the date of the last attended program session. All adverse events were recorded using researcher logs, including any adverse events not related to the GRIT intervention but which occurred during the study implementation or at home and were reported to GRIT staff members. Participant and parent/guardian satisfaction with the GRIT program were measured on separate hard copy surveys at study completion or withdrawal. The Likert-scaled questions related to satisfaction with the program overall, each discipline component and the staff involved (see Supplementary Materials pages 5–7).

#### Heart rate during exercise sessions

Prior to the GRIT intervention commencing, children and their parents were instructed in the proper set up of both their Polar watch and private Polar account. The HR data collected during exercise sessions was accessed via a central Polar Coach account. The participants were able to view their own exercise data when uploaded, but not the data of other involved participants. The uploaded HR data included beats per minute (bpm) measured at 00:01 s intervals. For each uploaded session (not including make-up sessions) the participants’ minimum, maximum, and mean HR were calculated. The child’s mean HR as a % of HRmax (as determined by their baseline cardiorespiratory testing data, see below) was then calculated for each session. For all uploaded exercise sessions, each of these HR data measures were averaged across the children. The data for sessions within weeks 2–4 (week 1 set up/ familiarisation period excluded), weeks 5–7, and weeks 9–12 were then each averaged for assessment of trends across the exercise program phases. Uploaded HR data was also used to determine completion of make-up sessions.

#### Anthropometry

Outcome measures were collected at baseline (0–3 weeks pre-intervention) and follow-up (up to 3-weeks post-intervention; i.e. 12–15 weeks post-baseline). Anthropometric measures were performed at baseline. Weight (kg) was measured using calibrated scales with light clothing, and shoes removed. Height (cm) was measured by a standing stadiometer using the stretch stature method. Body Mass Index (BMI, kg/m^2^) was calculated. Waist circumference (cm) was measured using a tape measure at the narrowest point between the lower ribs and the iliac crest. All anthropometric measures were repeated twice with the average of the two measures used as the outcome. However, if these measures differed by 5% or more a third measure was taken, and the average of the two closest measures was reported. BMI-for-age percentiles according to sex were determined [37] and used to calculate BMI Z-scores which classified participants as thin (<− 1), healthy (− 1 to + 1), overweight [1, 2] or obese (> 2); although research is ongoing regarding the language used to describe these categories.

#### Maximal graded cardiorespiratory testing

Cardiorespiratory testing was performed at baseline and follow-up at a local physiotherapist clinic. This type of exercise testing assesses ventilatory gas exchange in order to measure metabolic functional capacity [38]. Participants performed a resting test and treadmill ramp protocol with respiratory gas analysis (Ultima CPX™ metabolic stress testing system, MGC diagnostics) and a facemask system (preVent® Face Mask, MGC Diagnostics). The tests were implemented by trained clinical physiotherapists and flow and gas calibration were performed on the machine as per manufacturer instructions prior to each test. Participants were instructed to perform the test in a fasted (at least 6 h) and rested state (no exercise that day prior to the test). Where possible, time of day when the baseline and follow-up tests were performed was within 2 h. A self-report of usual exercise sessions undertaken per week was recorded at baseline and 12-weeks follow-up. Participants also undertook a resting metabolic test at baseline and 12 weeks (protocol detailed in Supplementary Materials).

The metabolic stress testing system calculated breath-by-breath measures of oxygen uptake (VO_2_) and carbon dioxide output (VCO_2_) with HR (bpm) also measured continuously during the test. Maximal exercise capacity is typically measured by a levelling off of VO_2_ despite increased workload (VO_2_max). However, as reported in previous studies in children [39], it was anticipated that most participants would not reach a VO_2_max. Instead, the peak oxygen consumption (VO_2_peak) was chosen to be reported. VO_2_peak was calculated as the average of the two highest VO_2_ measures recorded during the test. Other outcomes included: exercise test duration (time in minutes and seconds between start of test and volitional exhaustion); the testing time at which VO_2_peak was reached (average of the times at which the two highest VO_2_ measures occurred); HR at the start of exercise testing; maximum HR measured (HRmax, average of the two highest HR measures recorded during the test); and the testing time at which HRmax was measured (average of the times at which the two highest HR measures occurred).

The breath-by-breath gas analysis recorded from both the resting tests (the mean of the last 10-min of data) and exercise tests (means of the data from each 1-min testing increment from 10-min onwards) were also used to measure substrate oxidation. Based on the calculated respiratory quotient (VCO_2_/VO_2_), fat and carbohydrate oxidation and energy expenditure were calculated using stoichiometric equations and appropriate energy equivalents, with the assumption that the urinary nitrogen excretion rate was negligible during the treadmill test [36, 40]. Maximum fat oxidation (MFO) [41] was calculated in kcal/min based on the highest fat oxidation measure within the 1-min testing increments calculated during the exercise test. MFO time was also recorded as the 1-min testing increment within which the MFO measure occurred.

#### Nutrient intake and diet quality

Nutrient intake and diet quality were measured using the Australian Child and Adolescent Eating Survey (ACAES) at baseline and post-intervention. These were completed online where participants’ parent/guardians were emailed the survey link with instructions. The ACAES is a validated 135-item semi-quantitative food frequency questionnaire (FFQ) which reflects the Australian food supply, and includes 120 food items and 15 supplementary questions addressing demographics and food and activity behaviours [42]. Parents/guardians were encouraged to have their child complete the survey questions on their own as this has been reported to produce more accurate intake data [43]. The ACAES licence holders (University of Newcastle, Australia) performed analysis after final data collection. Data included estimated daily micro- and macronutrient intakes, contribution to total energy intake from food groups, and the Australian Child and Adolescent Recommended Food Score (ACARFS). The ACARFS is a validated food-based diet quality index which quantifies overall diet quality reflecting the level of adherence to the Australian Dietary Guidelines for children and adolescents [44]. The ACARFS has a total diet quality score ranging from 0 to 73 (73 indicating the highest possible diet quality); as well as eight sub-scales for the food groups of vegetables, fruit, grains, meat, meat alternatives, dairy, extras and water [44]. The ACARFS shows strong correlations with nutrient intakes; however, is independent of BMI for children and adolescents, indicating that improvements in dietary intake can be demonstrated without the requirement to consume more food (and energy by proxy) overall [44].

#### Psychological assessment: self-concept

The participants’ self-concept was assessed using the validated Piers-Harris Children’s Self-Concept Scale, 2nd Edition (Piers-Harris 2) [45]. This tool is suitable for children aged 7–18 years and takes 10–15 min to complete the 60 items. It evaluates the domains of behavioural adjustment, intellectual and school status, physical appearance and attributes, popularity, happiness and satisfaction, and freedom from anxiety [45]. This tool was self-completed by the participants in small group sessions facilitated by the psychologist at baseline and follow-up. The psychologist analysed the children’s completed Piers-Harris 2 forms according to standardised procedures in which raw scores were converted to standardised *T*-scores (mean = 50, standard deviation = 10). This resulted in a total score (general self-concept) and sub-scores for each of the previously noted six domains, with a higher score reflecting greater self-concept (refer to supplementary Table S2 for interpretation of the *T*-Score ranges). This tool also measured two scales that assessed the validity of the responses: inconsistent responding and response bias. A *T*-score ≥ 70 for the inconsistent responding scale suggested a child may have responded randomly to some questions and a *T*-score ≤ 30 or ≥ 70 for the response bias scale may represent a tendency toward negative or positive response bias, respectively [45].

### Statistical analyses

All statistical analyses were conducted in SPSS statistical package version 25 [IBM Corp, released 2018]. Statistical significance was set at *p* < 0.05. The Shapiro-Wilk test was applied to assess the normality of continuous variables. Data are presented as mean ± standard deviation (SD), median (interquartile range [IQR]), or n (%), as appropriate. An Independent Student’s *t*-test or non-parametric Mann-Whitney U test was used to compare continuous variables between study completers and dropouts at baseline, whereas categorical variables were compared using the *Chi-square* test. A Paired samples *t*-test or Wilcoxon signed-rank test was used to determine the effect of the intervention on continuous outcome variables between baseline and follow-up in study completers only (defined as participants who completed 12-week maximal exercise testing). Repeated measures ANOVA, with post-hoc *t*-tests, was used to assess differences in exercise HR data measures across weeks 2–4, 5–8 and 9–12 of the intervention. If data was missing for study completers at follow-up due to failure to complete/attend the measures, their data were primarily analysed (and reported herein) by bringing baseline observations forward as a conservative method which assumes no change [46]. Analyses in only study completers without missing data were also performed to confirm any impact of imputation on results for intervention effect.

## Results

### Participants

**Fig. 1:**
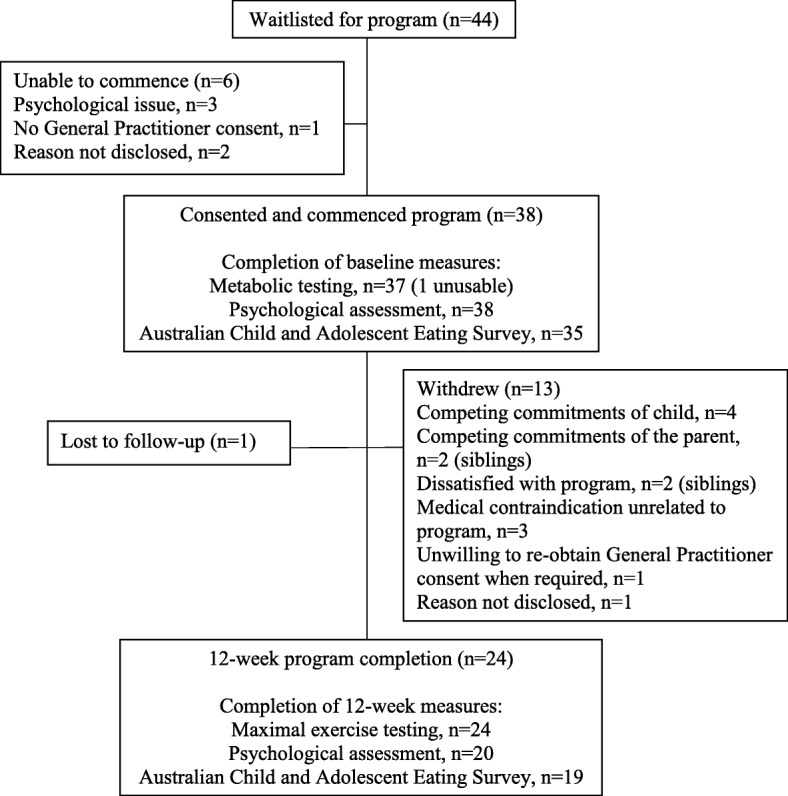
Flow diagram of participants in Project GRIT, including completion of study measures and reasons for withdrawal

A total of 44 potentially eligible participants were identified in the recruitment timeframe, six of whom were ineligible or unable to start the program (Fig. 1). Therefore, 38 eligible children and adolescents were recruited. At baseline, the total participant cohort median age was 11.4 years (range: 8.8 to 15.8 years), 53% were male, and 66% had BMI Z-score > 1 and median BMI percentile of 95 (IQR 47–98) (Table 4).

**Table 4:** Baseline characteristics of participants enrolled in GRIT (*n* = 38)

Measure	Total cohort
	*Median (IQR)*^*a*^*, n (%), or mean ± SD*
Age (years)	11.4 (9.7–12.9)
Male sex	20 (53)
Sibling involved	16 (42)
Weight (kg)	56.7 ± 18.7
Waist circumference (cm)	76.7 ± 13.7
BMI (kg/m^2^)	23.5 ± 5.5
BMI for age (%le)	95 (47–98)
BMI Z-score	1.6 (−0.10–1.99)
< −1	1 (42)
−1 to 1	12 (32)
> 1 to 2	16 (42)
> 2	9 (24)
Exercise sessions/week	2.0 (1.0–3.0)

*BMI* Body mass index

^*a*^Non-parametric data

### Process evaluation

Of the 38 enrolled participants, 24 (63%) completed the 12-week intervention. Withdrawals occurred from within week 1 to week 11 of the program (3 in week 1, 2 in week 2, 3 in week 3, 1 in week 4, 1 in week 6, 1 in week 7, 2 in week 10 and 1 in week 11); where the main reasons were competing commitments (*n* = 4) and medical contraindications unrelated to the intervention (*n* = 3) (Fig. 1). There were no significant differences between completers and dropouts for these general characteristics of participants at baseline (supplementary Table S3).

#### Program attendance

Attendance at GRIT sessions for all participants and completers are reported in Table 5. The program completers attended a median 58% (total range 30 to 88%) of the 33 offered exercise sessions. Dropouts attended a median 38% (total range 0 to 48%) of offered exercise sessions in the first 4 weeks and then a median of 0 thereafter (supplementary Table S4). Make up exercise sessions were completed for one quarter of the sessions missed by completers and no make-up sessions were done by dropouts. Completers attended a median 60% (total range 0 to 100%) of the 5 offered dietary sessions, compared to 20% (total range 0 to 40%) in dropouts. Only completers (42%) attended the one EFT/mindfulness psychologist session which occurred in week 8 of the program. Within the dropouts the mean time they were involved in the study was 27 ± 20 out of 82 program days.

**Table 5:** Attendance at Project GRIT program sessions, reported as median (IQR)

Measure	Total cohort (n = 38)		Completers (n = 24)	
	*No. sessions*	*% of offered*	*No. sessions*	*% of offered*
Exercise sessions (out of 33)^a^	17.5 (5.0–20.5)	53 (15–62)	19.0 (18.0–25.0)	58 (55–75)
Weeks 1–4 (out of 12)	8.5 (5.0–10.3)	71 (42–85)	9.0 (8.3–11.0)	75 (69–92)
Weeks 5–8 (out of 11)	6.0 (0–7.3)	55 (0–66)	7.0 (6.0–8.0)	64 (55–73)
Weeks 9–12 (out of 10)	2.0 (0–5.0)	20 (0–50)	4.5 (2.3–6.0)	45 (23–60)
Dietary sessions (out of 5)	2.0 (1.0–4.0)	40 (20–80)	3.1 (2.0–4.8)	60 (40–95)
EFT/Mindfulness session (out of 1)	n = 10	26%	n = 10	42%

*EFT* Emotional Freedom Technique (tapping)

^a^33 exercise sessions offered as 3 were cancelled (1 in week 5–8 and 2 in week 9–12)

#### Adverse events

No serious adverse events occurred. Minor adverse events which were self-reported by participants occurred during exercise sessions only and did not require medical intervention. On six occasions a participant started but did not complete an exercise session; four of these were possibly related to the intervention (sore knee, sore groin, sore leg and *n* = 2 feeling unwell), and one was not related to the intervention (recent stitches on finger). On another six occasions a participant reported an event but completed the exercise session; all were possibly related to the intervention (soreness or pain in a leg, knee, and/or heel). The quality of footwear (provided by the parents and not the study) was identified by staff as a frequent cause of minor adverse events related or possibly related to the intervention. Other minor events unrelated to the program were: 1) half way through the program, one parent reported being concerned that their child may have disordered eating habits and was referred by medical centre staff to an eating disorders specialist (externally) and the participant remained in the program but chose not to attend any further healthy eating workshops or cooking demonstrations; 2) a participant reported having had an asthma attack at school (not-exercise induced) and then discontinued the program as they were not willing to obtain General Practitioner re-consent.

#### Satisfaction surveys

Satisfaction surveys were returned by 12 parents and 15 participants. One participant and their parent who submitted the satisfaction surveys represented a dropout who withdrew from the program after 6-weeks and the remainder were completers. Satisfaction survey response data has been provided in detail in the Supplementary Table S5, including suggestions for potential improvements made by the survey respondents. Most participants (87%) and parents (83%) who responded reported they were very satisfied or satisfied with the GRIT program. Most of these participants (80%) and parents (75%) indicated they were also satisfied with the time spent in the GRIT program. For the participants, the proportion rating ‘very satisfied’ was highest for the cooking demonstrations (60%), followed by EFT/mindfulness (57%, in the 7 who had attended), exercise sessions (47%), and the healthy eating sessions (40%). Two thirds of both parents and participants who responded indicated they would ‘definitely’ recommend the program to a friend.

### Heat rate during exercise sessions

In the completers who had accessible HR data through their Polar account (*n* = 22 with a mean 21 ± 5 exercise sessions of data available per participant), there was a mean increase of 5 bpm maximum recorded HR across the program (*p* = 0.001) (Table 6). Mean HR as a % of the participants’ HRmax (from baseline maximal exercise testing data) slightly decreased across the program (*p* = 0.046), with a significant mean decrease of 2 bpm between weeks 2–4 and weeks 5–8 only (*p* = 0.002).

**Table 6:** Heart Rate (HR) data measured during exercise sessions in completers (n = 22)

HR Measure	Weeks 2-4^a^	Weeks 5–8	Weeks 9–12	
	*Mean ± SD*			*p-value*
Minimum	111 ± 11	110 ± 7	109 ± 9	0.23
Maximum	191 ± 8	193 ± 8	196 ± 9	0.001*
Mean	147 ± 9	144 ± 7	146 ± 8	0.06
Mean % HRmax	75 ± 5	73 ± 4	74 ± 5	0.046**

HRmax, estimated maximum heart rate from baseline maximal exercise testing data

^a^Week 1 set up / facilitation period excluded

*Significant difference in maximum recorded HR across the program (weeks 2–4 vs. 5–8, *p* = 0.08; weeks 2–4 vs. 9–12, p = 0.002; weeks 5–8 vs. 9–12, *p* = 0.02)

**Significant difference in % HRmax across the program, (weeks 2–4 vs. 5–8, p = 0.002, weeks 2–4 vs. 9–12, *p* = 0.29; weeks 5–8 vs. 9–12, *p* = 0.23)

### Study outcome measures

#### Maximal graded cardiorespiratory testing

**Fig. 2:**
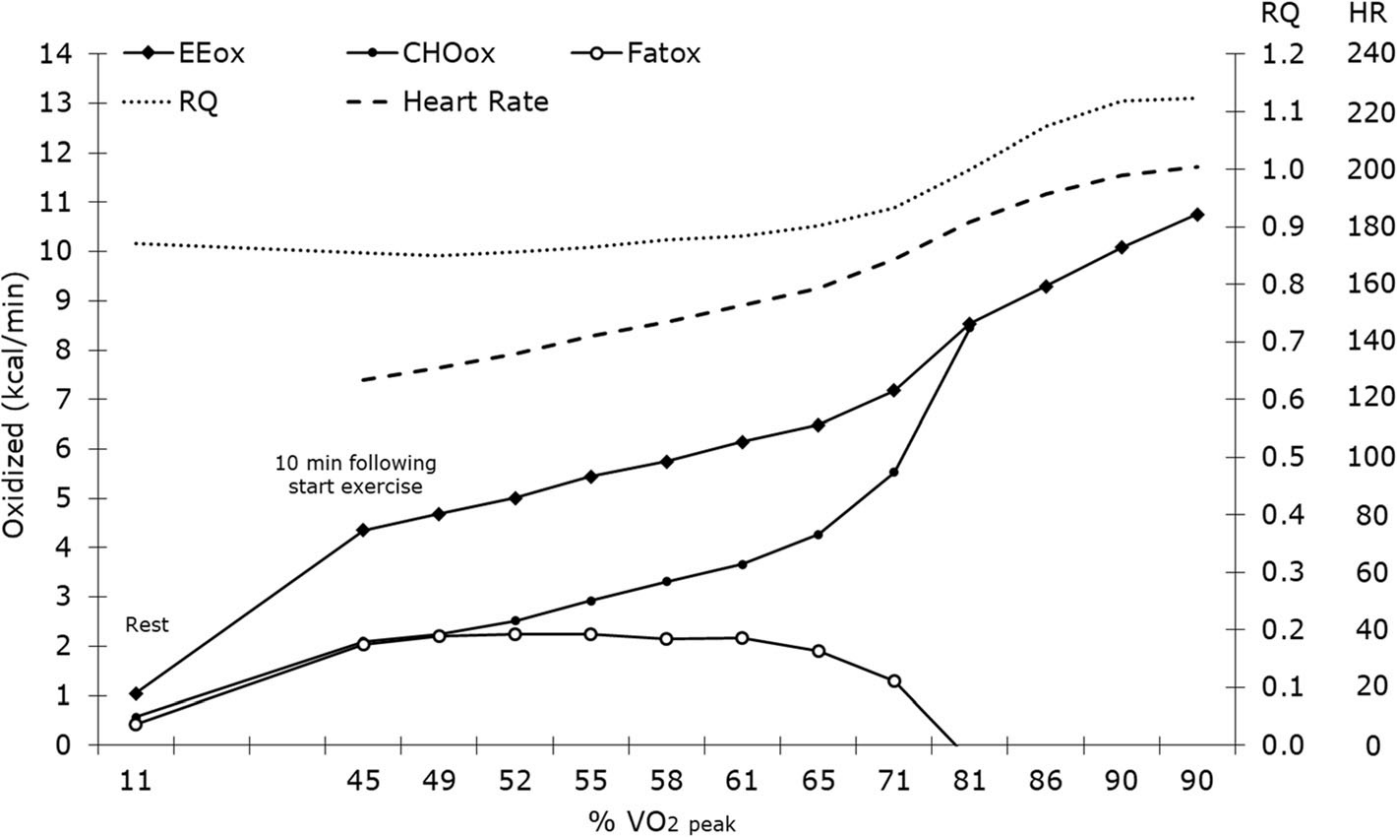
Substrate oxidation in function of VO2peak (%) during a graded treadmill test to exhaustion. EEox is the amount of total energy expenditure in kcal/min. CHOox is the amount of carbohydrate oxidized in kcal/min. Fatox is the amount of fat oxidized in kcal/min. RQ is the respiratory quotient calculated as the ratio of carbon dioxide (CO_2_) produced divided by oxygen (O_2_) consumed during the exercise. HR is heart rate in bpm. Data are means across all participants

Figure 2 illustrates the participants’ substrate oxidation and HR in function of their VO_2_peak at rest and in 1-min increments from 10-min in the maximal exercise test at baseline. Completers and dropouts did not differ in baseline maximal exercise test results (see supplementary Table S6).

Maximal exercise test outcomes for the 24 completers are reported in Table 7. There were no significant changes between baseline and 12-weeks; absolute VO_2_peak was, however, modestly increased by 5% (96 ± 239 mL/min) after 12-weeks when compared to baseline (*p* = 0.06).

**Table 7:** Maximal graded cardiorespiratory test outcomes in GRIT program completers (*n* = 24)

Measure	Baseline	12-weeks	
	*Mean ± SD or Median (IQR)*^*c*^		*p-value*
Test duration (min:sec)	19:41 ± 2:00	19:52 ± 01:09	0.63
VO_2_peak (absolute, ml/min)	1922 ± 469	2018 ± 468	0.06
VO_2_peak time (min:sec)	19:13 ± 02:06	19:32 ± 01:12	0.38
HR exercise start (bpm)	110 ± 14	111 ± 11	0.93
HRmax (bpm)	201 (192–205)	198 (192–203)	0.58
HRmax test time (min:sec)	19:19 ± 02:05	19:28 ± 01:05	0.67
^a^MFO (kcal/min)	2.7 ± 1.0	2.4 ± 0.9	0.12
^b^MFO time (1 min interval)	13 (11–15)	12 (10–17)	0.76

*HRmax* maximum recorded heart rate, *MFO* maximum fat oxidation

^a^MFO data for *n* = 23 due to errors in one participant testing

^b^MFO time represents the 1-min interval in the testing period at which peak fat oxidation occurred

^c^Non-parametric data

#### Nutrient intake and diet quality

Two dropouts and one completer did not complete their online ACAES at baseline. For the baseline dietary intake data collected, 86% were reported as being completed by the child and the remainder by a parent/guardian. Seven completers and two dropouts completed their baseline online eating survey late (after the first healthy eating session had occurred); however, their data has still been included as the eating survey asks questions relating to the past 3-months and inclusion would more likely reduce the reported effect on dietary improvement at follow-up than inflate it. At baseline, dropouts had higher diet quality compared to completers (see supplementary Table S7); specifically, total ACARFS (median 34.0 [IQR 27.0–45.5] vs. 23.0 [IQR 18.0–35.0], *p* = 0.03), vegetable ACARFS (mean 12.1 ± 5.7 vs. 7.3 ± 5.0, *p* = 0.01), and percentage vegetable contribution to energy intake (median 5.5 [IQR 5.0–9.5] vs. 4.0 [IQR 2.0–5.0] %, *p* = 0.009). With regards to nutrient intake, the dropouts had significantly higher daily intake of water (mean 2.8 ± 0.8 vs. 2.2 ± 0.7 L, *p* = 0.02) and vitamin C (median 155.9 [IQR 113.7–235.4] vs. 86.2 [53.0–264.8] mg, p = 0.01).

Four of the 23 completers who had baseline diet data did not complete their follow-up online ACAES, so their baseline data was carried forward to follow-up (Table 8). At follow-up, 89% of the surveys were reported as being completed by the participant and the remainder by a parent/guardian. There was an increase in mean percentage contribution to energy intake from total core foods (by 6.0 ± 11.1%, p = 0.02), accompanied by the same % reduction in energy intake from non-core foods, from baseline to follow-up data. This was contributed to by a decrease in median percentage contribution to energy from confectionary (− 2.0 [IQR 0.0–3.0] %, *p* = 0.003) and baked products (− 1.0 [IQR 0.0–5.0] %, *p* = 0.02). Although some improvements were reported for total and food group-based ACARFS and for nutrient intakes, none were statistically significant. The dietary intake results remained the same when analyses were performed in study completers with complete data only.

**Table 8:** Dietary intake outcomes in GRIT program completers (*n* = 23)

Measure	Baseline	12-weeks	
	*Mean ± SD or Median (IQR)*^*a*^		*p-value*
*Food Percentage Contribution to Daily Energy Intake*			
Core	55.7 ± 17.0	61.7 ± 12.4	0.02*
Non-core	44.3 ± 17.0	38.4 ± 12.4	0.02*
Vegetables	4.0 (2.0–5.0)	4.5 (2.0–7.0)	0.38
Fruit	7.8 ± 4.3	10.1 ± 7.4	0.12
Grains	15.0 (8.0–19.0)	15.5 (10.3–18.8)	0.86
Meat	12.6 ± 6.0	14.7 ± 7.8	0.21
Meat alternatives	2.0 (1.0–5.0)	2.0 (1.0–4.8)	0.64
Dairy	9.0 (8.0–17.0)	11.5 (7.0–22.0)	0.41
Sweet drinks	3.0 (1.0–5.0)	2.0 (1.0–4.8)	0.89
Packaged snacks	6.0 (3.0–10.0)	4.5 (3.0–9.8)	0.73
Confectionary	6.0 (4.0–12.0)	5.0 (4.0–8.8)	0.003*
Baked products	6.0 (4.0–9.0)	5.0 (3.0–7.0)	0.02*
Takeaway	9.0 (8.0–16.0)	10.0 (9.0–16.8)	0.78
Condiments	2.0 (1.0–3.0)	2.0 (1.0–3.0)	0.12
Fatty meats	2.0 (1.0–3.0)	2.0 (1.0–3.0)	0.23
*Australian Recommended Food Scores*			
Total (/73)	23.0 (18.0–35.0)	26.0 (19.3–39.5)	0.28
Vegetables (/21)	7.3 ± 5.0	6.8 ± 4.9	1.00
Fruit (/12)	4.0 (3.0–7.0)	5.0 (2.3–7.8)	0.25
Grains (/13)	4.6 ± 2.1	4.8 ± 2.5	0.13
Meat (/7)	2.3 ± 1.1	2.4 ± 1.6	0.73
Meat alternatives (/6)	1.0 (1.0–2.0)	2.0 (1.0–2.0)	0.79
Dairy (/11)	3.7 ± 2.2	4.0 ± 2.4	0.12
Extras (/1)	1.0 (1.0–2.0)	1.0 (1.0–2.0)	1.00
Water (/2)	1.0 (0.0–1.0)	1.0 (1.0–1.0)	0.66
*Daily Nutrient Intake*			
Energy (kJ)	9078 ± 2689	8663 ± 3507	0.52
Protein (g)	94.4 ± 28.8	96.7 ± 42.9	0.75
Protein (%E)	17.0 (16.0–19.0)	18.0 (17.0–21.8)	0.39
CHO (g)	252.3 ± 79.9	230.1 ± 93.4	0.23
CHO (%E)	47.7 ± 5.7	45.9 ± 8.6	0.31
Fat (g)	83.5 ± 28.5	80.9 ± 36.5	0.71
Fat (%E)	35.4 ± 4.8	35.3 ± 6.1	0.86
Saturated fat (g)	38.1 ± 14.7	35.9 ± 17.7	0.53
Saturated fat (%E)	15.9 ± 3.0	15.3 ± 3.4	0.52
PUFA (g)	8.9 ± 2.9	8.6 ± 4.1	0.93
PUFA (%E)	4.0 (3.0–4.0)	4.0 (3.3–4.0)	0.59
MUFA (g)	29.4 ± 9.9	27.4 ± 13.8	0.89
MUFA (%E)	12.4 ± 2.0	12.8 ± 2.5	0.52
Cholesterol (mg)	326.4 ± 114.4	303.0 ± 172.9	0.81
Sugars (g)	131.1 ± 55.8	105.4 ± 48.1	0.18
Water (L)	2.2 ± 0.7	2.1 ± 1.0	0.63
Fibre (g)	22.1 (22.2–29.9)	23.7 (15.3–30.6)	0.99
Vitamin C (mg)	86.2 (53.0–264.8)	110.2 (57.1–150.5)	0.40
Folate (μg)	235.0 (235.0–359.7)	256.9 (173.7–353.3)	0.86
Niacin (mg)	21.2 ± 6.7	21.2 ± 9.2	0.99
Niacin equivalents (mg)	40.1 ± 11.8	40.6 ± 17.5	0.86
Riboflavin (mg)	2.2 ± 0.9	2.2 ± 1.1	0.87
Thiamin (mg)	1.6 (0.8–1.8)	1.5 (0.9–2.1)	0.52
Vitamin A (μg)	1145.7 (802.5–1614.7)	1261.9 (771.1–1753.0)	0.71
Retinol (μg)	497.3 (306.5–632.8)	429.3 (429.3–700.0)	0.58
Beta-carotene (μg)	3694.0 (1974.7–5720.7)	4092.9 (1487.9–6383.2)	1.00
Sodium (mg)	2197.8 ± 702.1	2141.3 ± 999.0	0.76
Potassium (mg)	3093.0 ± 997.4	3104.2 ± 1306.4	0.96
Magnesium (mg)	332.4 ± 88.1	339.2 ± 130.4	0.75
Phosphorus (mg)	1578.1 ± 513.3	1589.6 ± 723.5	0.93
Iron (mg)	12.1 ± 3.7	12.1 ± 4.7	0.97
Zinc (mg)	12.3 ± 3.6	12.7 ± 5.5	0.62
Calcium (mg)	987.6 (713.7–1318.6)	906.5 (601.1–1695.9)	0.61

*CHO* carbohydrate, *PUFA* polyunsaturated fatty acids, *MUFA* monounsaturated fatty acids

^a^Non-parametric data

*Significant difference between baseline and follow-up, *p* < 0.05

#### Self-concept

Two dropouts did not complete the Piers-Harris 2 self-concept scale at baseline. There were no significant differences between dropouts and completers for total or individual domain scores (supplementary Table S8); however, dropouts did tend to have a lower score for the ‘Happiness and Satisfaction’ domain (41.5 [IQR 37.8–43.0) vs. 45.0 [IQR 40.0–51.0], *p* = 0.08). At baseline and follow-up, no participants had an inconsistent responding scale *T*-score ≥ 70. At baseline two completers had response bias scale *T*-scores of 29, which could reflect a tendency of negative reporting. No completers had a response bias score ≤ 30 at 12-weeks. No participants had a response bias scale *T*-score ≥ 70 at baseline or follow-up.

Four completers did not complete the Piers Harris-2 assessment at follow-up; hence their baseline scores were carried forward (Table 9). There was an improvement in total mean score (by 2.8 ± 5.3, *p* = 0.02) and the ‘Physical appearance and attributes’ area median score (by 4.0 [IQR 0.5–4.0], p = 0.02) from baseline to follow-up. The score for ‘Happiness and satisfaction’ also changed by a median score of 4.0 (IQR 0.0–8.0), *p* = 0.10. There were no changes in the scales for response bias and inconsistent reporting. The self-concept results remained the same when analyses were performed in only study completers with complete data.

**Table 9:** Piers-Harris 2 Self-concept Scale outcomes^a^ in GRIT program completers (*n* = 24)

Scale	Baseline	12-weeks^**b**^	
	*Mean ± SD or Median (IQR)*^*c*^		*p-value*
Total score	48.2 ± 9.4	51.0 ± 10.8	0.02*
Behavioural adjustment	54.0 (46.8–62.0)	54.0 (49.0–62.0)	0.30
Intellectual and school status	51.0 (48.0–54.0)	51.0 (46.0–54.0)	0.39
Physical appearance and attributes	42.0 (40.0–50.3)	48.0 (40.5–54.3)	0.02*
Freedom from anxiety	47.0 (37.0–54.0)	52.5 (37.0–58.0)	0.08
Popularity	47.0 ± 10.4	47.3 ± 9.8	0.62
Happiness and satisfaction	45.0 (40.0–51.0)	49.0 (40.0–59.0)	0.10
Response bias	49.2 ± 10.5	49.8 ± 9.6	0.85
Inconsistent responding	53.0 (43.0–53.0)	43.0 (43.0–53.0)	0.29

^a^A higher score represents better self-concept

^b^Baseline data carried forward for four participants who failed to complete 12-week assessment

^c^Non-parametric data

*Significant difference between baseline and follow-up, p < 0.05

## Discussion

The primary aim of this study was to determine the feasibility and effect of a multidisciplinary lifestyle intervention delivered in a non-institutional setting on cardiorespiratory fitness, nutrient intake, diet quality and self-concept in sedentary children and adolescents. The results demonstrated that study completers improved diet quality through an increased proportion of energy intake from healthy core foods and decreased discretionary foods, and improved self-concept, particularly with regards to the physical appearance and attributes domain. Cardiorespiratory fitness was not significantly improved at follow-up, although mean absolute VO_2_peak increased 5%; a comparable modest increase to previous intervention studies in children [47]. Despite being satisfied with the program, few recruited participants met the attendance goals and the attrition rate was higher than expected.

The GRIT program found no significant improvement in cardiorespiratory fitness, with trends demonstrated for increased absolute VO_2_peak and HR during exercise. It has previously been demonstrated in research settings that HIIT interventions for 5 and 12 weeks significantly improved cardiorespiratory fitness and cardiometabolic risk markers in children with similar effects to MICT [25, 26]. A recent randomised trial conducted in Australia of HIIT vs. MICT in children with obesity found a greater increase in cardiorespiratory fitness, as measured by relative VO_2_peak, with HIIT compared to MICT [39]. In that study the sessions were conducted individually in a controlled environment using an exercise bike, which is not reflective of a real-world setting. Our study was unique in testing the use of a HIIT protocol in children in a group setting and in a non-controlled environment. A randomised controlled feasibility study in New Zealand involving overweight inactive adults (*n* = 49) similarly assessed 12-weeks of supervised HITT group sessions held outdoors in a community park [48]. The intervention improved VO_2_max; however, the magnitude was more modest than demonstrated in prior adult trials. The authors concluded this was most likely due to the reduced adherence to the exercise program when moving beyond the research clinic setting, a phenomenon which was likely also experienced by GRIT participants.

Participants reduced intake of discretionary foods as a contribution to energy intake, with a significant reduction in confectionary and baked products, and increased total intake of healthy core foods. Whilst there was no significant increase in any individual healthy core food, there were small increases in each which contributed to the total improvement. Our program used predominantly experiential learning in the dietary education and cooking demonstration sessions, which aligns with previous findings that school-based programs for children with the most significant improvements for increased healthy food intake and reduced sugar intake used this delivery technique [21]. The same review identified that parental involvement in childhood healthy eating programs was associated with program effectiveness in the school setting [21]. Parents were encouraged to attend the GRIT dietary sessions, however, not all took part and the sessions were mostly directed at the children. Inclusion of more parent-focused dietary education could have been useful, which was also self-reported by some parents in the feedback surveys.

There was no significant improvement in the ACARFS as a measure of overall diet quality, the food group sub-scores or intake of nutrients in GRIT participants. The total and food group ACARFS are scored based on both total daily intake and the variety of choices within the food groups [42] to reflect the Australian Dietary Guidelines [31]. The GRIT healthy eating sessions and cooking demonstrations promoted healthy core food choices, including recipe modification and balance with non-core discretionary foods (which significantly improved), however, the education did not necessarily target increased variety of healthy foods eaten. Future programs could therefore benefit from greater emphasis on the importance of variety within core food groups.

Completers of GRIT had a significant improvement in Piers-Harris 2 total self-concept and physical appearance and attributes scores. This improvement in self-concept was most likely an impact of the exercise and dietary sessions as the psychology session which was held in the last month of the program was attended by less than one third of these participants. The GRIT program emphasised goals for healthy eating and physical activity rather than weight status and was delivered in a group setting, which are both strategies recommended for improving self-esteem in higher BMI children [20]. A previous group-based cognitive behavioural therapy, physical activity and dietary intervention in adolescents aged 13 to 16 years improved global self-perception and domains for physical appearance, social acceptance and romantic appeal [49]. On the other hand, a multidisciplinary group-based healthy eating and physical activity program involving children aged 6 to 12 years and their families only saw a significant improvement in participants with an initial BMI ≥98th percentile. A review concluded that improvements in self-concept or self-esteem from exercise interventions in children and adolescents were likely linked to attainment of skills and addition of activities (rather than replacement) [50]. Having recruited participants with mostly low baseline activity levels, it is likely that the GRIT program achieved both.

Attendance at program sessions was lower than the expected target of 80% and over one third of participants withdrew from the program. Other studies of lifestyle intervention in children and adolescents in Australia, have reported a similar drop-out rate [39, 51]. The aforementioned Australian trial of HIIT versus MICT in children with obesity had higher exercise session attendance rates (average 68%); however, it demonstrated a similar trend to GRIT for reduction in attendance rates across its 12-week program [39]. The other previously noted study which assessed the feasibility of HIIT in adults in a real-world setting had an attendance rate of 59% in their aerobic interval training group, which is similar to the attendance rates at exercise sessions for GRIT completers. Competing commitments were the main reason for dropout in GRIT which is a difficulty associated with delivering an intervention outside of the school setting. Availability of parent/guardians to supervise the exercise sessions may have also impacted attendance. Feedback from parents included holding dietary education on the same days as the exercise to reduce the number of days involved in the week and to avoid having the program run during school holidays. Furthermore, feedback from both GRIT participants and parents highlighted that the dietary sessions could have been improved by dividing participants based on age groups. Delivering and targeting dietary activities for age groups could increase attendance at those sessions as well as their effectiveness.

At baseline, the study dropouts had significantly higher total ACARFS and vegetable sub-score, and tended to engage in more exercise, compared to completers. This suggests that participants who followed a healthier lifestyle may have been less engaged with the program. Whilst the study was intended to enrol only sedentary children, this was self-reported by their parent/guardian and no screening tool was used. It is recommended that exercise levels be assessed via a valid tool to identify sedentary children and adolescents in future programs. Future programs may also be better targeted at inclusion of children and adolescents identified as having poor diet quality at baseline, or the inclusion of some individualised sessions (as was suggested in the parent’s feedback) could assist with identifying targeted areas of dietary improvement for each participant.

This pilot study is strengthened by testing a multidisciplinary intervention in a real world, non-institutional setting. Furthermore, it was unique in not being focused on weight and including children of any BMI. However, this study was limited by not having a control group and therefore cause and effect cannot be concluded regarding the observed improvements in diet quality and self-concept. An FFQ validated for Australian children and adolescents was used to measure dietary intake, however the results may be limited by self-reported data. As a pilot study, the participant numbers were small and outcome measures were not powered to detect a significant change. Whilst the process evaluation will be helpful in informing the design of future programs, the satisfaction surveys distributed to participants who withdrew were poorly completed and the recommendations generated are mostly limited to the opinions of participants and parents who completed the study.

## Conclusions

The 12-week Project GRIT pilot indicated promising results; the group-based multidisciplinary lifestyle intervention for children and adolescents in a non-institutional setting improved diet quality and self-concept in study completers. A lack of significant improvement in cardiorespiratory fitness may have been impacted by declining attendance rates at exercise sessions across the program. Future practice and research should focus on providing sustainable multidisciplinary lifestyle interventions for children and adolescents with a focus on those identified as having poor dietary and physical activity habits, parental involvement and incorporating flexibility to enhance engagement.

## Supplementary information

**Additional file 1.** Supplementary Materials including: healthy eating and cooking demonstration session details; parent and participant satisfaction surveys, results tables reporting baseline participant characteristics, program attendance and baseline data for exercise testing, dietary intake and self-concept scale of study completers versus dropouts; and additional GRIT satisfaction survey data.

## Data Availability

The datasets used and/or analysed during the current study are available from the corresponding author on reasonable request.

## Abbreviations

ACAES: Australian Child and Adolescent Eating Survey
ACARFS: Australian Child and Adolescent Recommended Food Score
APD: Accredited Practising Dietitian
BMI: Body Mass Index
BPM: Beats per minute
CHO: Carbohydrate
CHOox: Carbohydrate oxidation
EEox: Total energy expenditure
EFT: Emotional Freedom Technique
Fatox: Fat oxidation
FFQ: Food frequency questionnaire
GRIT: Growth, Resilience, Insights, Thrive
HIIT: High Intensity Interval Training
HR: Heart rate
HRmax: Maximum recorded heart rate
IQR: Interquartile range
MFO: Maximum fat oxidation
MICT: Moderate Intensity Continuous Training
MUFA: Monounsaturated fatty acids
PUFA: Polyunsaturated fatty acids
SD: Standard deviation
VCO2: Carbon dioxide output
VO2: Oxygen consumption
VO2max: Maximal exercise capacity
VO2peak: Peak oxygen consumption
