# Supplementary material for: Multidisciplinary lifestyle intervention in children and adolescents - results of the project GRIT (Growth, Resilience, Insights, Thrive) pilot study
Source: BMC Pediatr. 2020 Apr 20;20:174. doi: 10.1186/s12887-020-02069-x (PMC7169025; doi:10.1186/s12887-020-02069-x)
Supplement: Supplementary file 1 — Additional file 1. Supplementary Materials including: healthy eating and cooking demonstration session details; parent and participant satisfaction surveys, results tables reporting baseline participant characteristics, program attendance and baseline data for exercise testing, dietary intake and self-concept scale of study completers versus dropouts; and additional GRIT satisfaction survey data. [file 12887_2020_2069_MOESM1_ESM.pdf]

Multidisciplinary lifestyle intervention in children and adolescents - results of the Project GRIT (Growth, Resilience, Insights, Thrive) pilot study

Hannah L Mayr, Felicity Cohen, Elizabeth Isenring, Stijn Soenen, Project GRIT Team and Skye Marshall

SUPPLEMENTARY MATERIALS

Table S1 Details of the GRIT Program Healthy Eating and Cooking Demonstration Sessions

Session details	Session content	Resources used
Healthy Lunchbox challenge<br>Healthy Eating Session 1<br>Week 1<br>Held by APD at WLSA offices<br>30-minute duration<br>2 session options offered	Example core and discretionary food samples were provided<br>In pairs the children selected food options for their school lunchbox and shared these choices with the group<br>Group discussion: which foods were fresh versus processed<br>Children were asked to demonstrate the fresh options they had chosen in their lunchbox<br>Group discussions:<br>• the benefits of eating fresh foods (e.g. nutrients, lasting energy)<br>• identification of other healthy/fresh options in the food sample options not chosen by pairs<br>• identification of foods which were processed/discretionary options<br>• achieving balance in food choices<br>Children returned chosen food items and then in pairs re-chose their lunch box to include more fresh items, including some balance of discretionary foods and shared their change in choices with the group	Real food samples including core foods and discretionary food items (e.g. fruit, nuts, muesli bars, packaged savoury snacks, sandwiches, canned fish)

	Group discussions:<br>• involvement in making lunch for school<br>• changes children could make to increase fresh options and improving balance of foods<br>• sharing of other fresh lunch options not included in the samples	
Healthy Snack Recipe Modification<br>Healthy Eating Session 2<br>Week 3<br>Held by APD at WLSA offices<br>30-minute duration<br>2 session options offered	APD recapped previous session on choosing fresh foods and balance in your diet<br>and outlined activity for this session: chocolate lamington ball recipe modified to healthy fruit and nut balls<br>Children tasted raw ingredients, almonds and dried dates, individually and discussed:<br>• texture, flavour and look<br>• use as snacks or ingredients in cooking and baking<br>Children provided with prior blended ingredients (almonds, dates, coconut and cocoa powder) to roll into small balls and coat in desiccated coconut<br>Group discussions:<br>• what is the fourth ingredient? (cocoa powder)<br>• other commercial chocolate products with ingredients such as added sugar versus this recipe which uses natural ingredients including healthy fats and natural sugar from fruit<br>• other similar fresh / healthy ingredient options that could be used to modify recipes<br>Children tasted the fruit and nut balls and packed the remainder to take home and try with parents and/or siblings<br>Children provided with a copy of recipe and encouraged to make as a snack at home	Raw almonds and dried dates for tasting<br>Combined almonds, dried dates, desiccated coconut and cocoa powder in a food processer and extra desiccated coconut (individually packed in containers for participants).<br>Plastic sandwich bags for storing balls to take home<br>Recipe cards
Guest Chefs<br>Cooking demonstration 1<br>Week 5	Chef 1 – Cooking demonstration of breakfast meal: scrambled eggs and avocado rye toasty<br>Chef 2 – Cooking demonstration of snack: healthy rocky road<br>Both demonstrations involved discussion of healthy ingredients and cooking techniques<br>After each demonstration children and parents were able to trial samples of the completed meal or snack	Chefs for demonstration<br>Commercial kitchen and cooking equipment

Held by 2 local Guest Chefs at local commercial kitchen<br>1-hour duration<br>1 session offered, with children rotating between two cooking stations 20-minutes each	Electronic copy of recipes provided	Food ingredients<br>Recipe booklet
Food for Mood<br>Healthy Eating Session 3<br>Week 6<br>Held by APD at WLSA offices<br>30-minute duration<br>2 session options offered	A series of magazine cut-outs of a variety of pictures of foods and meals displayed on the floor<br>Children and parents asked to find a food or meal picture that they associate with a happy memory<br>Group discussion: children and parents shared why their chosen food or meal picture is a happy memory for them<br>APD led discussions:<br>• summarised the key themes associated with reasons for food and happy memories<br>• the importance of considering how foods make you feel as well as the nutrition they provide, including sharing of meals and social eating<br>• Recapped key points from prior healthy eating sessions<br>Group discussions:<br>• the children's feelings in relation to the guest chef cooking demonstration<br>• positive feelings when involved in cooking and food preparation<br>Hard copy of the Australian Dietary Guidelines, Healthy Eating for Children brochure provided	Magazine cut-outs of a variety of meals and snacks,<br>Healthy Eating for Children brochures
Healthy Sushi Rolls<br>Cooking Demonstration 2<br>Week 10	All pre-cooked and prepared sushi ingredients displayed on table<br>Group discussions:<br>• who has eaten sushi before, who likes sushi, who has made sushi before	Healthy sushi ingredients (vegetable sticks,

Held by APD at WLSA offices with assistance from project administrator<br>30-minute duration<br>2 session options offered	• what ingredients are commonly used in sushi<br>• Eating sushi as a meal or snack<br>• Healthy ingredient choices for sushi<br>APD demonstrated how to combine ingredients and roll sushi<br>All children with assistance from APD, project administrator and attending parents made their own sushi rolls and ate together<br>Group discussions:<br>• the process of making sushi and tips for improving rolls<br>• making sushi at home and where to get the ingredients and utensils required	brown rice, seaweed rolls, reduced salt soy sauce, sushi seasoning, shredded pre-cooked chicken breast)<br>Sushi rolling utensils (bamboo mats, cling wrap, plates and bowls for the ingredients)

APD, Accredited Practicing Dietitian; WLSA, Weight Loss Solutions Australia

GRIT Participant Satisfaction Survey

We are interested in your satisfaction of your experience in Project GRIT. Please circle which word that best indicates the way you feel.

1. How satisfied were you with the exercise sessions?

Very satisfied   Satisfied   Not satisfied or dissatisfied   Dissatisfied   Strongly dissatisfied

2. How satisfied were you with the healthy eating sessions?

Very satisfied   Satisfied   Not satisfied or dissatisfied   Dissatisfied   Strongly dissatisfied

3. How satisfied were you with the cooking demonstrations?

Very satisfied   Satisfied   Not satisfied or dissatisfied   Dissatisfied   Strongly dissatisfied

4. How satisfied were you with the mindfulness session?

Very satisfied   Satisfied   Not satisfied or dissatisfied   Dissatisfied   Strongly dissatisfied

5. How satisfied are you with the amount of time spent doing Project GRIT activities?

Very satisfied   Satisfied   Not satisfied or dissatisfied   Dissatisfied   Strongly dissatisfied

6. Overall, how satisfied are you with Project GRIT?

Very satisfied   Satisfied   Not satisfied or dissatisfied   Dissatisfied   Strongly dissatisfied

7. Did you feel that the staff listened to you?*

Always   Sometimes   Not sure   Not really   Never

8. Did you feel the staff responded to your needs?*

Always   Sometimes   Not sure   Not really   Never

9. Did you feel the staff gave you enough support?*

Always   Sometimes   Not sure   Not really   Never

10. Would you recommend participating in Project GRIT to your friends?

Definitely   Maybe   Not sure   Not really   Never

11. Do you have any ideas to make Project GRIT better? (write your ideas below)

*Results not reported for these questions which relate to personnel involvement.

GRIT Parent Satisfaction Survey

We are interested in your satisfaction of your child's experience in Project GRIT. Please circle which word that best indicates the way you feel.

1. How satisfied were you with the exercise sessions?

Very satisfied Satisfied Not satisfied or dissatisfied Dissatisfied Strongly dissatisfied

2. How satisfied were you with the healthy eating sessions?

Very satisfied Satisfied Not satisfied or dissatisfied Dissatisfied Strongly dissatisfied

3. How satisfied were you with the cooking demonstrations?

Very satisfied Satisfied Not satisfied or dissatisfied Dissatisfied Strongly dissatisfied

4. How satisfied were you with the mindfulness session?

Very satisfied Satisfied Not satisfied or dissatisfied Dissatisfied Strongly dissatisfied

5. How satisfied are you with the amount of time spent doing Project GRIT activities?

Very satisfied Satisfied Not satisfied or dissatisfied Dissatisfied Strongly dissatisfied

6. Overall, how satisfied are you with Project GRIT?

Very satisfied Satisfied Not satisfied or dissatisfied Dissatisfied Strongly dissatisfied

7. Did you feel that the staff listened to you?*

Always Sometimes Not sure Not really Never

8. Did you feel the staff listened to <u>your child</u>?*

Always Sometimes Not sure Not really Never

9. Did you feel the staff responded to your needs?*

Always Sometimes Not sure Not really Never

10. Did you feel the staff responded to <u>your child's</u> needs?*

Always Sometimes Not sure Not really Never

11. Did you feel the staff gave <u>your child</u> enough support?*

Always Sometimes Not sure Not really Never

12. Would you recommend participating in Project GRIT to your friends / friend's children?

Definitely   Maybe   Not sure   Not really   Never

13. Do you have any ideas to make Project GRIT better? (write your ideas below)

*Results not reported for these questions which relate to personnel involvement.

Protocol for the Resting Metabolic Testing procedure at baseline and 12-weeks

The resting test was performed for 15-minutes with the participant in a seated position and in front of an age appropriate television program, to minimise spontaneous activity and to distract them from the testing apparatus. Parents were nearby in the waiting area, but not visible, during the resting test. For the exercise test the child was instructed to exercise to their maximal limit and was fitted with a heart rate monitor chest strap (Polar, T31). The participants were asked whether or not they agreed to be encouraged late in the test, if so, this was repeated during the follow-up test. Parents were invited to stay in the room; however, were asked to sit behind the subject (out of sight) during the testing procedure. Participants completed a 4-minute warm up at 4 km/hr while they were familiarised with the treadmill walking. Following the warm up, the gradient was increased to 2% for 3 minutes, then it was further increased to 4% for another 3 minutes. The fourth stage was 1-minute duration, continuing at 4 km/h but with 6% gradient. From there the incline was increased by 2% every additional minute up to a maximum gradient of 18%. If this maximum gradient was reached the treadmill speed was increased by 1 km/hr each additional minute until volitional exhaustion was reached (and the child stopped the treadmill). Beyond a respiratory exchange ratio (RER) of 0.9 participants who had agreed to encouragement were encouraged by the physiotherapist every 20 seconds. Subjects that showed signs of high effort prior to reaching RER of 0.9 were encouraged earlier. Post-testing, subjects were asked to keep the face mask on for 1-2 minutes to continue data collection during recovery.

Table S2 Interpretation of Piers-Harris 2 *T*-Scores*

T-Score Range	Percentile Range	Interpretative Label
Total Scale		
≤29*T*	≤2	Very low
30-39*T*	3-14	Low
40-44*T*	15-28	Low Average
45-55*T*	29-71	Average
56-59*T*	72-83	High Average
60-69*T*	84-97	High
≥70*T*	≥98	Very High
Domain Scales		
≤29*T*	≤2	Very low
30-39*T*	3-14	Low
40-44*T*	15-28	Low Average
45-55*T*	29-71	Average
≥56*T*	≥72	Above Average

*Adapted from Piers & Herzberg. (2002). Piers-Harris Children's Self-Concept Scale(2nd ed.) (Piers-Harris 2) [Manual]. Torrence, CA: Western Psychological Services.

Table S3 Baseline characteristics of study completers versus dropouts

**Measure**	**Completers (n=24)**	**Dropouts (n=14)**	
	Median (IQR), n (%) or mean ± SD		*p-value*
Age (years)	11.3 (9.6-12.1)	11.7 (10.3-14.6)	0.24
Gender, Male	12 (50)	8 (57)	0.67
Sibling involved	8 (33)	8 (57)	0.15
Weight (kg)	56.1 ± 18.2	57.8 ± 20.1	0.77
Waist circumference (cm)	76.6 ± 13.5	77.0 ± 14.7	0.93
BMI (kg/m^2)	23.4 ± 5.6	23.7 ± 5.5	0.85
BMI for age (%le)	95 (33-97)	94 (55-98)	0.89
BMI Z-score	1.6 (-0.4-1.9)	1.6 (0.1-2.1)	0.85
<-1	-	1 (7)	
-1 to 1	7 (29)	5 (36)	
>1	17 (71)	8 (57)	
Exercise sessions/week	1.5 (1.0-3.0)	2.5 (1.0-4.3)	0.31

BMI, Body Mass Index

Table S4 Program attendance in study dropouts, reported as median (IQR)

**Measure**	**Dropouts (n=14)**	
	No. sessions	*% of offered*
Exercise sessions (out of 33)*	4.5 (2.5-9.3)	14 (8-14)
Week 1 to 4 (out of 12)	4.5 (2.5-5.8)	38 (21-38)
Week 5 to 8 (out of 11)	0 (0.0-0.5)	0 (0-0)
Week 9 to 12 (out of 10)	0 (0.0-0.0)	0 (0-0)
Diet sessions (out of 5)	1.0 (0-1.25)	20 (0-25)
EFT/Mindfulness session (out of 1)	n=0	0%

EFT, Emotional Freedom Technique (tapping)

*33 exercise sessions offered as 3 were cancelled (1 in week 5-8 and 2 in week 9-12)

Table S5 Project GRIT Satisfaction survey data completed by 12 parents and 15 participants[a]

	**Exercise sessions**	**Healthy eating sessions**	**Cooking demonstr-ations**	**EFT / mindfulness session***	**Time spent in program**	**GRIT overall**
12 parents						
Very satisfied	6	2	6	2	5	5
Satisfied	4	7	5	2	4	5
Neutral	1	2	1	0	3	2
Dissatisfied	1	1	0	1	0	0
Very dissatisfied	0	0	0	0	0	0
15 children						
Very satisfied	7	6	9	4	7	10
Satisfied	5	5	2	2	5	3
Neutral	3	3	3	0	2	2
Dissatisfied	0	1	0	1	1	0
Very dissatisfied	0	0	0	0	0	0

EFT, Emotional Freedom Technique (tapping)
[a]Data are frequency of responses and includes 1 parent and child who dropped out at 6-weeks, the remainder were study completers
*Represents data from 5 parents and 7 participants who attended this session

Summary of open-ended feedback or suggestions for improvements from participants and parents completed surveys

Children:

- Include a get to know you game
- Motivate kids more
- Improve healthy eating activities
- Have more hands-on activities that are suitable per age group
- Feeling healthier, fitter and smarter after the program

Parents:

- Include goal setting for something to work towards
- Consideration of working parent demands, business hours activity challenges
- Run program within school terms
- Closer to home would make it easier
- Have dietary sessions at the end of exercise sessions to avoid an extra day of commitment
- Mix up exercises from just running, include more variation and make more engaging
- Activity tracker/watch was difficult to set up
- Focus on parent education to encourage behaviour changes, particularly diet and participation/commitment
- More cooking demonstrations with the kids involved would be great
- Provide a meal plan
- Include individual diet checks/ food sessions
- Divide healthy eating sessions by age group, some of the activities were boring for the older kids
- Greater selection of foods for the pack your own lunch session
- Provide a recipe book with recipes that the kids could cook
- Provide/suggest post program options for continued fitness/lifestyle programs

Table S6 Baseline maximal graded cardiorespiratory testing data of study completers versus dropouts

Measure	Completers (n=24)	Dropouts (n=13)[a]	
	Mean ± SD or median (IQR)		*p-value*
Test duration (min:sec)	19:41 ± 2:00	20:33 ± 01:41	0.47
VO2 peak (absolute, ml/min)	1922 ± 469	2184 ± 732	0.19
VO2 peak time (min:sec)	19:13 ± 02:06	19:54 ± 01:31	0.31
HR exercise start (bpm)	110 ± 14	107 ± 154	0.47
HRmax (bpm)	201 (192-205)	197 (189-206)	0.56
HRmax test time (min:sec)	19:19 ± 02:05	19:52 ± 01:25	0.43
FATmax (kcal/min)	2.7 ± 1.0	2.9 ± 1.3	0.66
[b]FATmax time	13 (11-15)	13 (11-15)	0.52

HRmax, maximum recorded heart rate; FATmax, maximum fat oxidation
[a]Exercise testing data reported for dropouts n=13 as one dropout requested to stop test early (at 08:21)
[b]FATmax time represents the 1-minute interval in the testing period at which peak fat oxidation occurred

Table S7 Baseline dietary intake data of study completers versus dropouts

Measure	Baseline (n=23)	Dropouts (n=12)	
	Mean ± SD or Median (IQR)		*p-value*
Food Percentage Contribution to Daily Energy Intake			
Core	55.7 ± 17.0	58.5 ± 11.7	0.61
Non-core	44.3 ±17.0	41.5 ± 11.7	0.61
Vegetables	4.0 (2.0-5.0)	5.5 (5.0-9.5)	0.009
Fruit	7.8 ± 4.3	9.8 ± 4.0	0.18
Grains	15.0 (8.0-19.0)	16.0 (8.3-21.3)	0.84
Meat	12.6 ± 6.0	11.8 ± 6.9	0.73
Meat alternatives	2.0 (1.0-5.0)	3.5 (1.3-5.0)	0.36
Dairy	9.0 (8.0-17.0)	11.0 (2.5-20.0)	0.88
Sweet drinks	3.0 (1.0-5.0)	2.0 (0.0-5.5)	0.55
Packaged snacks	6.0 (3.0-10.0)	5.0 (3.0-8.3)	0.59
Confectionary	6.0 (4.0-12.0)	7.0 (3.3-10.3)	0.93
Baked products	6.0 (4.0-9.0)	6.5 (2.3-10.5)	0.75
Takeaway	9.0 (8.0-16.0)	10.0 (8.0-15.0)	0.85
Condiments	2.0 (1.0-3.0)	2.0 (1.0-4.0)	0.67
Fatty meats	2.0 (1.0-3.0)	1.0 (0.0-1.0)	0.09
Australian Recommended Food Scores			
Total (/73)	23.0 (18.0-35.0)	34.0 (27.0- 45.5)	0.03*
Vegetables (/21)	7.3 ± 5.0	12.1 ± 5.7	0.01*
Fruit (/12)	4.0 (3.0-7.0)	6.0 (3.5-8.8)	0.13
Grains (/13)	4.6 ± 2.1	5.4 ± 1.7	0.23
Meat (/7)	2.3 ± 1.1	2.3 ± 1.6	0.90
Meat alternatives (/6)	1.0 (1.0-2.0)	2.0 (1.3-3.0)	0.33
Dairy (/11)	3.7 ± 2.2	4.5 ± 2.5	0.30
Extras (/1)	1.0 (1.0-2.0)	1.5 (0.3-2.0)	0.40
Water (/2)	1.0 (0.0-1.0)	1.0 (0.3-1.0)	0.20
Daily Nutrient Intake			
Energy (kJ)	9078 ± 2689	8830 ± 3651	0.82
Protein (g)	94.4 ± 28.8	97.0 ± 46.6	0.86
Protein (%E)	17.0 (16.0-19.0)	18.0 (16.0-21.8)	0.58

CHO (g)<br>CHO (%E)	252.3 ± 79.9<br>47.7 ± 5.7	248.0 ± 106.5<br>48.3 ± 5.5	0.89<br>0.75
Fat (g)<br>Fat (%E)	83.5 ± 28.5<br>35.4 ± 4.8	76.4 ± 32.9<br>33.5 ± 3.3	0.51<br>0.23
Saturated fat (g)<br>Saturated fat (%E)	38.1 ± 14.7<br>15.9 ± 3.0	33.1 ± 15.3<br>14.4 ± 1.4	0.36<br>0.11
PUFA (g)<br>PUFA (%E)	8.9 ± 2.9<br>4.0 (3.0-4.0)	9.3 ± 4.4<br>4.0 (3.0-5.0)	0.78<br>0.50
MUFA (g)<br>MUFA (%E)	29.4 ± 9.9<br>12.4 ± 2.0	27.0 ± 11.3<br>12.0 ± 1.8	0.53<br>0.39
Cholesterol (mg)	326.4 ± 114.4	308.9 ± 163.2	0.71
Sugars (g)	131.1 ± 55.8	132.4 ± 59.2	0.95
Water (L)	2.2 ± 0.7	2.8 ± 0.8	0.02*
Fibre (g)	22.1 (22.2-29.9)	25.6 (20.8-36.1)	0.28
Vitamin C (mg)	86.2 (53.0-264.8)	155.9 (113.7-235.4)	0.01*
Folate (µg)	235.0 (235.0-359.7)	314.8 (314.8-364.0)	0.36
Niacin (mg)	21.2 ± 6.7	20.9 ± 9.5	0.89
Niacin equivalents (mg)	40.1 ± 11.8	40.1 ± 18.7	0.99
Riboflavin (mg)	2.2 ± 0.9	2.4 ± 1.4	0.70
Thiamin (mg)	1.6 (0.8-1.8)	1.5 (1.0-2.2)	0.81
Vitamin A (µg)	1145.7 (802.5-1614.7)	1105.7 (737.6-1876.6)	0.95
Retinol (µg)	497.3 (306.5-632.8)	393.1 (270.3-556.1)	0.31
Beta-carotene (µg)	3694.0 (1974.7-5720.7)	4353.1 (2950.4-8059.8)	0.49
Sodium (mg)	2197.8 ± 702.1	2213.1 ± 1086.7	0.96
Potassium (mg)	3093.0 ± 997.4	3543.2 ± 1455.8	0.29
Magnesium (mg)	332.4 ± 88.1	387.1 ± 127.3	0.15
Phosphorus (mg)	1578.1 ± 513.3	1671.3 ± 813.2	0.68
Iron (mg)	12.1 ± 3.7	12.9 ± 5.3	0.63
Zinc (mg)	12.3 ± 3.6	12.7 ± 5.9	0.79
Calcium (mg)	987.6 (713.7-1318.6)	1211.8 (818.8-1642.5)	0.28

CHO, carbohydrate; PUFA, polyunsaturated fatty acids; MUFA, monounsaturated fatty acids
*Significant difference between completers and dropouts, $p<0.05$

Table S8 Baseline Pier Harris-2 Self-concept Scale data of study completers versus dropouts

**Scale**	**Baseline (n=24)**	**Dropouts (n=12)[a]**	
	Mean ± SD or Median (IQR)		*p-value*
Total score	48.2 ± 9.4	44.0 ± 11.7	0.25
Behavioural adjustment	54.0 (46.8-62.0)	51.5 (42.3-60.0)	0.34
Intellectual and school status	51.0 (48.0-54.0)	39.0 (29.8-59.0)	0.30
Physical appearance and attributes	42.0 (40.0-50.3)	42.5 (38.0-51.0)	0.59
Freedom from anxiety	47.0 (37.0-54.0)	39.5 (35.0-47.5)	0.24
Popularity	47 ± 10.4	44.8 ± 10.7	0.60
Happiness and satisfaction	45.0 (40.0-51.0)	41.5 (37.8-43.0)	0.08
Response bias	49.2 ± 10.5	51.6 ± 9.9	0.56
Inconsistent responding	53.0 (43.0-53.0)	53.0 (45.5-60.0)	0.09

[a]Two participants did not complete the Piers Harris-2 at baseline
